# Cavitary Lesions and Pleural Effusion: A Case of Necrotizing Pneumonia in a Young Child

**DOI:** 10.7759/cureus.80272

**Published:** 2025-03-08

**Authors:** Amrita A Gujar, Aaska Patel, Louisdon Pierre

**Affiliations:** 1 Pediatrics, The Brooklyn Hospital Center, Brooklyn, USA

**Keywords:** pediatric pulmonology, pediatrics, pneumonia, pulmonology, tb

## Abstract

Necrotizing pneumonia is a rare and severe complication of bacterial pneumonia in children, characterized by cavitation and potential respiratory failure. A three-year-old child presented with fever, vomiting, and right-sided crackles on lung auscultation. Imaging revealed right upper lobe consolidation, and laboratory evaluation showed leukocytosis (WBC 29K), hyponatremia (Na 130 mEq/L), and elevated CRP (215.9 mg/L). Despite the use of intravenous antibiotics, the patient developed respiratory distress and required admission to the pediatric intensive care unit. Ultrasound revealed a loculated pleural effusion. The patient underwent video-assisted thoracic surgery with thoracotomy and chest tube placement. Cultures and acid-fast bacillus testing were negative, but pleural fluid analysis was consistent with exudative effusion. A CT scan revealed a cavitary lesion, confirming necrotizing pneumonia. He improved with antibiotics and surgical drainage and was discharged on a prolonged course of oral antibiotic regimen. Necrotizing pneumonia poses diagnostic challenges, particularly in cases with negative cultures. This case underscores the importance of a multimodal diagnostic approach, including imaging and pleural fluid analysis, to guide management. Multidisciplinary management, including antibiotics and surgical intervention, is critical for treating necrotizing pneumonia in pediatric patients.

## Introduction

Necrotizing pneumonia (NP) is a severe and potentially life-threatening complication of bacterial pneumonia characterized by liquefactive necrosis of lung parenchyma, cavitation, and systemic inflammation. It represents an advanced stage of pneumonia where lung tissue undergoes extensive necrosis, often leading to prolonged hospitalizations, significant morbidity, and potential long-term pulmonary complications. While NP remains relatively rare, its incidence has been increasing, possibly due to rising antimicrobial resistance and increased pathogen virulence [1,2].

Children with NP often present with nonspecific respiratory symptoms, including fever, cough, and tachypnea, which may rapidly progress to severe respiratory distress. The most commonly implicated pathogens include *Streptococcus pneumoniae*, *Staphylococcus aureus* (including methicillin-resistant *Staphylococcus aureus* {MRSA}), and *Pseudomonas aeruginosa*. In pediatric cases, early diagnosis can be challenging due to the overlapping clinical presentations of bacterial pneumonia, viral infections, and tuberculosis, especially in regions with a high burden of infectious diseases [3,4].

Radiological imaging, particularly contrast-enhanced computed tomography (CT), plays a crucial role in diagnosing NP by identifying characteristic findings such as pulmonary cavitation, pleural effusion, and parenchymal necrosis. However, the diagnosis is often complicated by the difficulty in obtaining microbiological confirmation, as blood cultures and pleural fluid cultures frequently yield negative results [5,6].

This report details the case of a three-year-old boy diagnosed with NP complicated by cavitation and pleural effusion, necessitating surgical intervention and prolonged antimicrobial therapy. The case highlights the diagnostic challenges posed by NP, particularly in the context of an indeterminate tuberculosis (TB) screening and social determinants such as recent immigration and limited access to follow-up care. The importance of early identification and comprehensive management of NP has been increasingly emphasized in recent literature, with studies highlighting long-term pulmonary outcomes and the role of corticosteroids in treatment strategies [7-12].

## Case presentation

A previously healthy three-year-old boy presented to the ED with a history of fever for five days (Tmax 103.2°F), non-bloody, non-bilious emesis, headaches, and body aches. He tolerated PO intake, with normal wet diaper output. The mother denied respiratory, gastrointestinal, or systemic symptoms and reported no recent travel or sick contacts. The patient recently immigrated from Ecuador in January 2024 and lived in a shelter.

**Figure 1:**
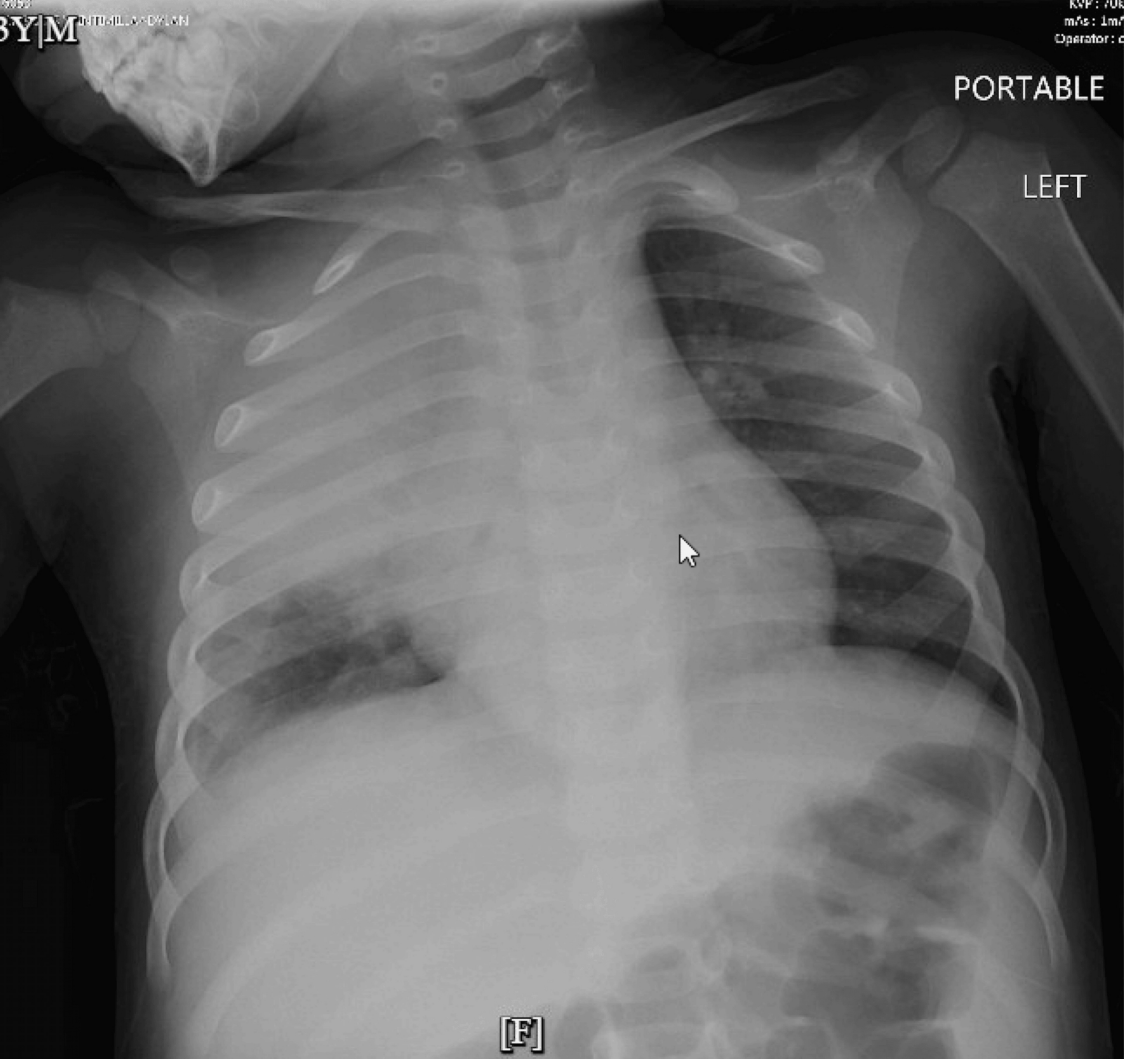
Chest X-ray, hospital day 1; dense opacity of the right mid to upper hemithorax may represent loculated pleural effusion, pneumonia, and/or atelectasis.

In the ED, the patient was febrile (102.3°F, 39.1°C) with normal vital signs: heart rate 105 beats per minute, respiratory rate 22 breaths per minute, oxygen saturation (SpO2) 96%, blood pressure (BP) 97/53 mm of Hg. Examination revealed crackles in the right upper lung field. Chest X-ray (CXR) showed right mid-to-upper lobe consolidation concerning pneumonia (Figure 1).

Labs revealed leukocytosis (WBC 29,000/µL, absolute neutrophil count {ANC} 24,500/µL), hyponatremia (Na 130 mEq/L), mild metabolic acidosis (bicarbonate 17 mEq/L), and elevated CRP (215.9 mg/L) (Table 1). Viral panel including respiratory syncytial virus (RSV), Covid, and Flu sent resulted negative. He received IV ceftriaxone, a normal saline bolus, and was admitted for pneumonia with presumptive bacteremia.

**Table 1:** Laboratory values on hospital day 1. ANC: absolute neutrophil count.

Laboratory test	Result	Reference range
WBC	29,000/µL	4,500-11,000/µL
ANC	24,500/µL	1,500-8,000/µL
Sodium (Na)	130 mEq/L	135-145 mEq/L
Bicarbonate	17 mEq/L	22-29 mEq/L
CRP	215.9 mg/L	<10 mg/L

**Figure 2:**
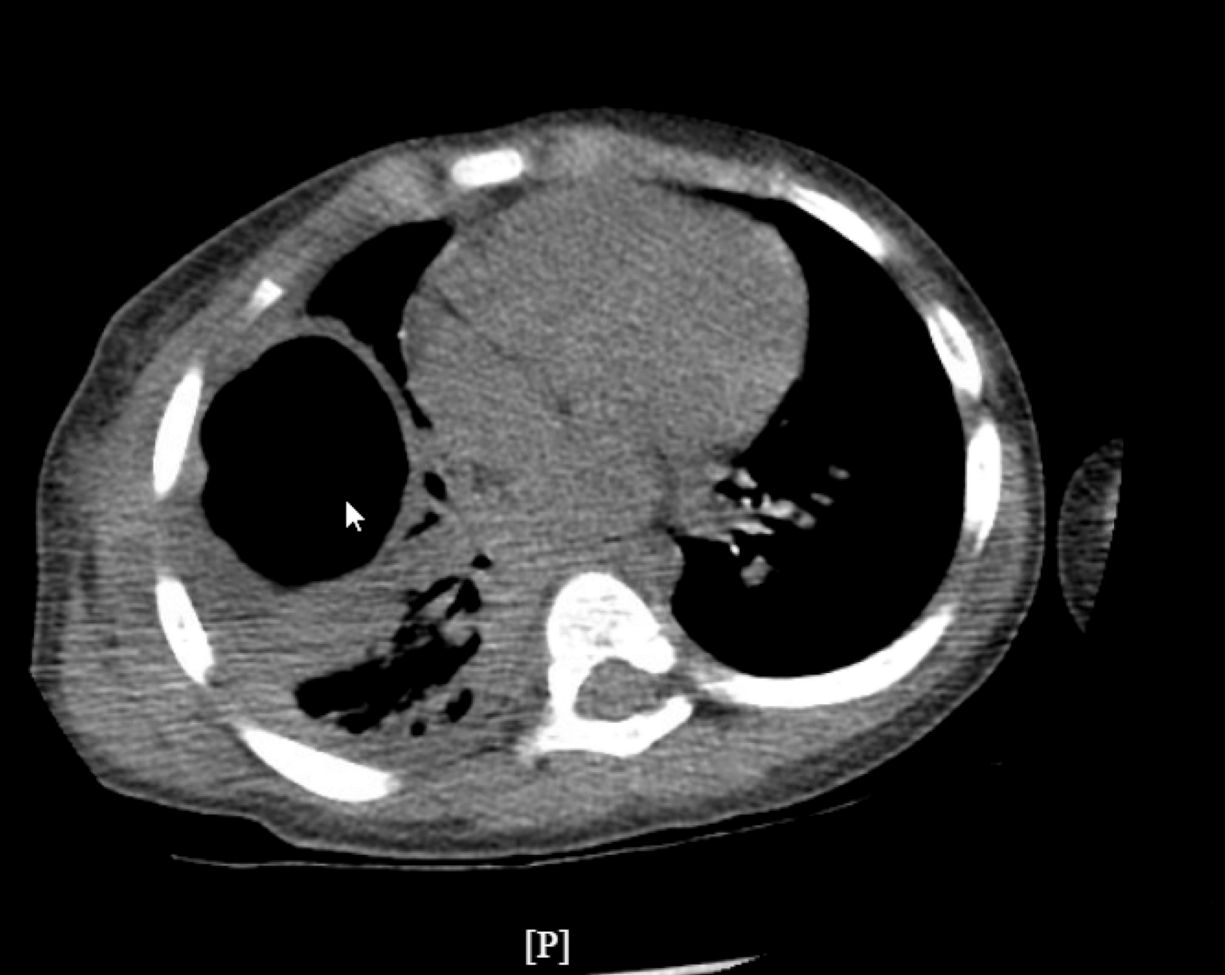
CT chest (transverse view), hospital day 1; large thick-walled cavity in the right upper lobe with surrounding smaller cavitary lesions.

**Figure 3:**
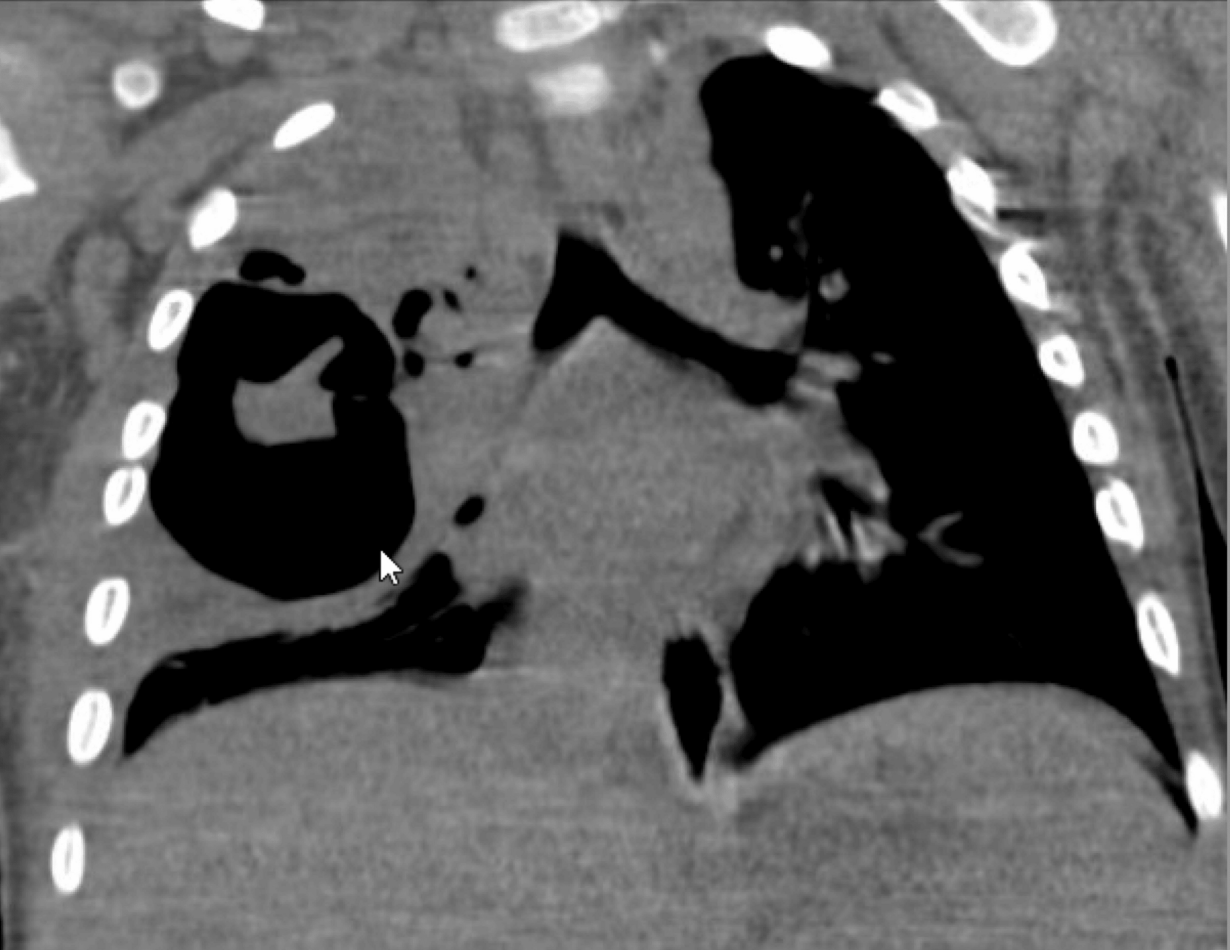
CT chest (coronal view), hospital day 1; large thick-walled cavity in the right upper lobe with surrounding smaller cavitary lesions.

On hospital day 2, the patient developed respiratory distress and was transferred to the pediatric intensive care unit (PICU) for non-invasive respiratory support. Chest ultrasound showed a loculated pleural effusion. Due to the suspicion of loculated effusion, he underwent a video-assisted thoracic surgery (VATS), converted to open thoracotomy with chest tube placement. Pleural fluid studies were consistent with an exudative effusion (WBC 75/µL, pleural lactate dehydrogenase {LDH} >3325 U/L, pleural albumin 2.1 g/dL). Cultures, including acid-fast bacillus (AFB), were negative. Postoperatively, the patient was managed on invasive mechanical ventilation, transitioned to high-flow nasal cannula (HFNC), and subsequently weaned to room air by hospital day 11. Antibiotics initially included only ceftriaxone on admission; however, on upgrading the patient to PICU on hospital day 2, vancomycin and azithromycin were added due to increased respiratory support requirement. Patient completed a five-day course of azithromycin and received ceftriaxone for 10 days and vancomycin for seven days before transitioning to oral augmentin to complete a three-week course for necrotizing pneumonia identified on CT (Figures 2, 3).

A follow-up Quantiferon test was indeterminate, and gastric lavage AFB cultures were negative for *Mycobacterium*. Pediatric pulmonology recommended outpatient follow-up and extended antibiotics.

**Figure 4:**
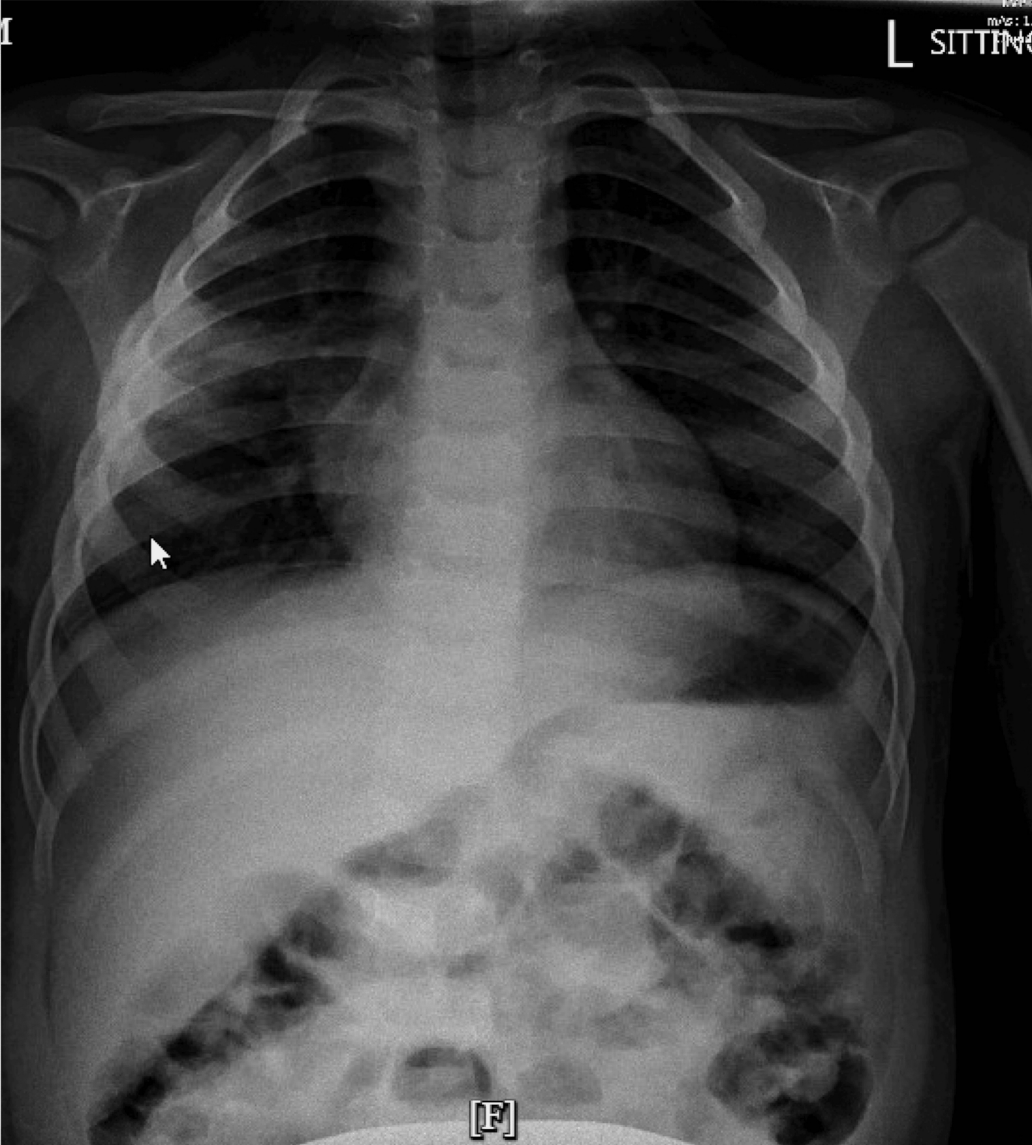
Chest X-ray, three months post admission; right pleural effusion and hazy opacities along the lateral aspect of the right lung, with decreased opacity since prior examination.

The patient returned to the ED one month after discharge from the hospital with fever. CXR showed improvement in the pneumatocoel (Figure 4). He was managed with supportive care without antibiotics and discharged.

## Discussion

Necrotizing pneumonia (NP) is a rare but severe complication of bacterial pneumonia, marked by pulmonary parenchymal necrosis, cavitation, and significant morbidity [1]. Pediatric patients are at higher risk for rapid progression to complications like pleural effusion, respiratory distress, and septic shock [2]. Common pathogens include *Streptococcus pneumoniae*, *Staphylococcus aureus *(including MRSA), and *Klebsiella pneumoniae*, although microbiologic confirmation is often elusive [3]. This diagnostic uncertainty complicates targeted therapy, necessitating a reliance on clinical features and imaging.

This case illustrates the diagnostic complexities of NP, particularly when cultures and tuberculosis testing are repeatedly negative. Imaging modalities, including CXR, ultrasound, and CT, were critical in identifying cavitation and guiding surgical intervention [2]. Pleural fluid analysis confirmed an exudative effusion, supporting the diagnosis of necrotizing pneumonia despite the absence of pathogen isolation [3]. This highlights the importance of integrating clinical, radiologic, and laboratory findings when microbiologic studies are inconclusive. Similarly, a study by Stultz et al. (2021) emphasized the diagnostic challenges and management strategies in children with necrotizing pneumonia, underscoring the importance of early recognition and appropriate interventions [4].

The patient required a multidisciplinary approach, with early escalation to PICU care and coordination between pulmonology, infectious diseases, and surgery. Surgical intervention via VATS and open thoracotomy was pivotal in managing the loculated pleural effusion and improving clinical outcomes [1]. Prolonged antibiotic therapy with ceftriaxone, azithromycin, and vancomycin was essential in addressing possible bacterial pathogens, and careful weaning from respiratory support was critical in ensuring recovery. Donadio et al. (2019) also highlighted the importance of timely surgical intervention and extended antibiotic regimens to address pediatric NP, particularly in complicated cases such as pleural effusion [5].

Additionally, Kolar et al. (2020) provided a detailed overview of the surgical management of necrotizing pneumonia, noting that early surgical intervention can significantly improve outcomes by draining loculated pleural fluid and addressing pulmonary cavitation [6]. Prolonged antibiotic therapy remains the cornerstone of treatment, with the appropriate antibiotics tailored to the suspected pathogens, as shown in this case where a three-week course of oral antibiotics was prescribed following discharge. Chen et al. (2023) also stressed the role of early identification and comprehensive treatment in improving outcomes for children with necrotizing pneumonia, particularly in settings with limited resources and challenging diagnostic conditions [7]. Moreover, Ae et al. (2020) suggested that delayed or inappropriate antibiotic use increases the risk of long-term pulmonary complications in pediatric NP cases, further emphasizing the necessity of early aggressive management [8].

Recent studies have also highlighted the impact of viral co-infections in the development of necrotizing pneumonia. Diaz-Diaz et al. (2019) demonstrated that preceding viral infections, such as influenza and RSV, predispose children to severe bacterial superinfections, increasing the risk of necrotizing pneumonia [9]. The immunologic dysregulation caused by these viral infections may contribute to the progression of pneumonia to its necrotizing form.

Furthermore, emerging evidence suggests a potential role of adjunctive therapies in pediatric NP. While antibiotic therapy and surgical interventions remain the mainstays of treatment, corticosteroids have been explored as an adjunctive therapy to reduce inflammation and lung damage. A systematic review by Ozola et al. (2023) indicated that corticosteroid use in select cases may be beneficial in improving outcomes, though further studies are needed to establish standardized guidelines [10].

Immunological factors also play a crucial role in NP progression. Children with underlying immunodeficiencies, including chronic granulomatous disease and primary antibody deficiencies, are at higher risk for severe necrotizing infections [11]. In cases where patients experience recurrent or unusually severe infections, an immunologic workup should be considered.

Additionally, long-term follow-up is critical in children recovering from NP, as studies have shown an increased risk of post-infectious bronchiectasis and restrictive lung disease. A longitudinal study by Herskovitz et al. (2022) found that nearly 25% of pediatric NP survivors exhibited persistent lung function abnormalities six months post-recovery, underscoring the need for pulmonary follow-up and rehabilitation when necessary [12].

Necrotizing pneumonia requires a high index of suspicion in pediatric patients with severe pneumonia and pleural effusions. Early surgical consultation and imaging are essential when conservative management fails, and prolonged antibiotic therapy remains the cornerstone of treatment [3]. This case demonstrates the importance of a multidisciplinary approach and highlights the need to address social determinants of health in complex pediatric conditions. Furthermore, recent guidelines emphasize the importance of long-term pulmonary follow-up and rehabilitation strategies to optimize recovery and prevent complications in survivors of pediatric NP [12].

## Conclusions

This case highlights the complex diagnostic and therapeutic challenges associated with necrotizing pneumonia in pediatric patients. Despite repeated negative microbiologic studies, imaging and pleural fluid analysis played pivotal roles in confirming the diagnosis and guiding management. The patient’s clinical course underscores the importance of early recognition, multidisciplinary collaboration, and timely surgical intervention in managing severe pneumonia complicated by pleural effusion and cavitation.

Additionally, this case emphasizes the impact of social determinants of health, such as recent immigration and resource constraints, on continuity of care and follow-up. Ensuring access to appropriate follow-up and addressing barriers is essential to optimize long-term outcomes. Necrotizing pneumonia remains a rare but life-threatening condition, and this report reinforces the importance of a systematic, individualized approach in its diagnosis and management.
